# A meta-analysis evaluating indirectly GLP-1 receptor agonists and arrhythmias in patients with type 2 diabetes and myocardial infarction

**DOI:** 10.3389/fcvm.2022.1019120

**Published:** 2022-10-05

**Authors:** Zhijie Liu, Ning Bian, Shaorong Wu, Yiming Fan, Hairui Li, Jian Yu, Jun Guo, Dongdong Chen

**Affiliations:** Department of Cardiology, The First Affiliated Hospital of Jinan University, Guangzhou, China

**Keywords:** Glucagon-Like Peptide 1 Receptor agonists, GLP-1RAs, arrhythmia, type 2 diabetes mellitus, myocardial infarction

## Abstract

**Aims:**

At present, the effects of Glucagon-Like Peptide 1 Receptor agonists (GLP-1RAs) on arrhythmia in patients with type 2 diabetes mellitus (T2DM) and myocardial infarction (MI) are still unclear. Hence, this systematic review and meta-analysis aimed to investigate this association.

**Methods and results:**

PubMed, Embase, Cochrane Library, and Web of Science were searched from inception to 30 April 2022. Randomized controlled trials (RCTs) that compared GLP-1RAs with placebo and met the critical criterion of a proportion of patients with T2DM and MI > 30% were included to verify our purpose indirectly. The outcomes of interest included atrial arrhythmias, ventricular arrhythmias, atrioventricular block (AVB), sinus arrhythmia, and cardiac arrest. Relative risk (RR) and 95% confidence intervals (CI) were pooled using a random-effects model. We included five RCTs with altogether 31,314 patients. In these trials, the highest proportion of patients with T2DM and MI was 82.6%, while the lowest was 30.7%. Compared to placebo, GLP-1RAs were associated with a lower risk of atrial arrhythmias (RR 0.81, 95% CI 0.70–0.95). There was no significant difference in the risk of ventricular arrhythmias (RR 1.26, 95% CI 0.87–1.80), AVB (RR 0.95, 95% CI 0.63–1.42), sinus arrhythmia (RR 0.62, 95% CI 0.26–1.49), and cardiac arrest (RR 0.97, 95% CI 0.52–1.83) between groups.

**Conclusion:**

GLP-1RAs may be associated with reduced risk for atrial arrhythmias, which seems more significant for patients with T2DM combined with MI. More studies are needed to clarify the definitive anti-arrhythmic role of this drug.

## Introduction

Cardiovascular disease (CVD) remains the most common cause of death in most countries worldwide, which is largely caused by the epidemic of type 2 diabetes mellitus (T2DM), according to the cardiovascular disease statistics 2021 of the European Society of Cardiology (1). Diabetes mellitus is identified as a coronary heart disease (CHD) risk equivalent, and many people with diabetes mellitus have a combination of myocardial infarction (MI) (2, 3). For them, the occurrence of arrhythmias, especially atrial fibrillation (AF), is a risk factor for developing heart failure and even cardiovascular death (4).

Glucagon-Like Peptide 1 Receptor agonists (GLP-1RAs) exert effect by simulating natural incretin Glucagon-Like Peptide 1 (GLP-1). At the same time, GLP-1RAs are not susceptible to degradation by dipeptidyl peptidase IV in the body. Accordingly, The hypoglycemic efficacy of GLP-1RAs acting on related receptors to promote insulin secretion is stable (5). As a novel hypoglycemic agent, GLP-1RAs have not only successfully undergone the cardiovascular outcomes trials (CVOTs) required by the United States Food and Drug Administration (FDA), but have also shown cardiovascular benefit in the evaluation results (6). At present, there are inconsistencies in the conclusions of previous studies regarding the relationship between GLP-1RAs and arrhythmias. The result of a longitudinal cohort study showed that the use of GLP-1 analogs was an independent risk factor associated with a higher incidence of atrial fibrillation (AF) (7). A meta-analysis evaluating cardiovascular safety of albiglutide found that more patients had AF or atrial flutter (AFL) in the experimental arm than in the compared arm (8). By contrast, a network meta-analysis showed GLP-1RAs could reduce the risk of AF/AFL in patients with diabetes compared with other glucose-lowering agents (9). Poudyal observed fewer arrhythmic episodes in those patients with CHD who received GLP-1 therapy during the perioperative period of coronary artery bypass grafting (10). Besides, some studies reported their neutral relationship. Both Al-Sadawi et al.’s (11) and Boulmpou et al.’s (12) meta-analyses indicated that treatment with GLP-1RAs does not significantly affect the risk for atrial or ventricular arrhythmias.

Some scholars have previously proposed a statement of differences in the overall design, participant population, and the like of the trials to explain the different effects of GLP-1RAs on cardiovascular outcomes in various studies (13). However, the underlying drivers of the variable results between GLP-1RAs and arrhythmias remain unknown. We speculate that the efficacy of GLP-1RAs on arrhythmias may be inconsistent in T2DM patients combined with different disease states, which may be one of the explanations for the heterogeneous results across the clinical studies described above.

Several previous preclinical studies have been conducted to validate the effects of GLP-1RAs on cardiac arrhythmias under the disease state of MI. Chen et al. found that treatment with exendin-4 reduced atrial fibrosis and inhibited atrial arrhythmias in the rat model of MI (14). Another animal study demonstrated that exendin-4 administered 1 h before ligation of the left anterior descending coronary artery in rats significantly prevented the development of ventricular arrhythmias after ischemia, such as reducing the incidence of ventricular fibrillation (VF) and the duration and number of ventricular tachycardia (VT) and VF episodes (15). Nevertheless, the effects of GLP-1RAs on arrhythmias in patients with T2DM and MI remain uncertain. Hence the objective of this systematic review and meta-analysis was to evaluate this association.

## Materials and methods

### Search strategy

We conducted and reported this systematic review and meta-analysis following the Preferred Reporting Items for Systematic Review and Meta-Analyses (PRISMA) 2020 statement (16). PubMed, Embase, Cochrane Library, and Web of Science from inception to 30 April 2022 were searched for eligible studies. In addition, reference lists of eligible articles and prior systematic reviews were manually searched for eligible studies. We performed our search using a combination of Medical Subject Headings terms and text words. The search strategy is shown in Supplementary Table 1.

### Selection criteria

We analyzed the population characteristics of the studies included in the above two meta-analyses with neutral results by searching and reading the original texts or their Supplementary material. One meta-analysis included 27 randomized controlled trials (RCTs), of which most used MI as an exclusion criterion, i.e., did not include the T2DM people combined with MI (11). In another meta-analysis, although all seven RCTs included patients with MI, the proportions varied considerably, e.g., 82.6% of such patients in the ELIXA study but only 16.2% in the REWIND study (12).

To be able to analyze indirectly the effect of GLP-1RAs on arrhythmias in patients with T2DM combined with MI, we set the proportion of patients with MI > 30% in the study population as a key inclusion criterion. In addition, studies fulfilling the following criteria were included: (1) RCTs that assessed the efficacy or safety of GLP-1RAs in patients with T2DM; and (2) reported at least one type of arrhythmias such as atrial arrhythmias, ventricular arrhythmias, or other as outcome events or serious adverse events (SAEs) in the original text, Supplementary material, or ClinicalTrials.gov. In order to ascertain the actual anti-arrhythmic effects of GLP-1RAs, we excluded those trials that treated patients with combination or non-placebo therapy. There were no restrictions on follow-up duration, language, publication date, or publication status.

### Data extraction and quality assessment

We used a pre-specified form to extract the following information: first author or trial name, year of publication, medication regimen, median follow-up duration, sample size, mean age, sex, number and proportion of MI patients, and ClinicalTrials.gov unique identifier. The outcomes of interest in our study include AF, AFL, atrial tachycardia (AT), ventricular fibrillation (VF), ventricular tachycardia (VT), ventricular extrasystoles (VE), atrioventricular block (AVB), sinus arrhythmia, and cardiac arrest. The above data were obtained from ClinicalTrail.gov, the original trial publication, or its Supplementary material. Following the method recommended in the Cochrane Handbook, we combined the multiple arms of different doses of the same drug in the same trial into a single arm (17).

The methodological quality of the RCTs was assessed by Cochrane’s Collaboration tool, including seven domains of random sequence generation, allocation concealment, blinding of participants and personnel, blinding of outcome assessment, incomplete outcome data, selective reporting, and others for assessing the risk of bias (18). Each domain was judged separately as “high risk,” “low risk,” or “unclear.” For each trial, the overall risk of bias was assessed as low risk if all domains were judged as low risk, as high risk if any domain was considered high risk, or otherwise as unclear.

Two investigators (Z.L. and Y.F.) independently performed study selection, data extraction, and quality assessment. Disagreements were resolved by consultation with the senior author (D.C.).

### Statistical analyses

The outcome of interest in this systematic review and meta-analysis was the incidence of arrhythmias. For dichotomous data, we selected relative risk (RR) and their 95% confidence intervals (CI) to summarize the effect estimates in the results. The statistical difference between GLP-1RAs and placebo was considered significant only for *p*-value ≤ 0.05, and RR < 1 would favor GLP-1RAs over placebo. Statistical heterogeneity across studies was assessed using the I^2^ statistic. An I^2^ < 50% was considered low statistical heterogeneity, and ≥ 50% was considered high. A random-effects model was used. The RevMan Version 5.4 (The Nordic Cochrane Centre, The Cochrane Collaboration, 2020) was used for all statistical analysis. In addition, subgroup analysis based on the drug classes of GLP-1RAs was performed. In the sensitivity analysis, we transformed the analysis to the fixed-effects models and used the odds ratio (OR) as the effect measure. We did not assess the publication bias as the small number of included studies (<10) in this analysis.

## Results

**FIGURE 1:**
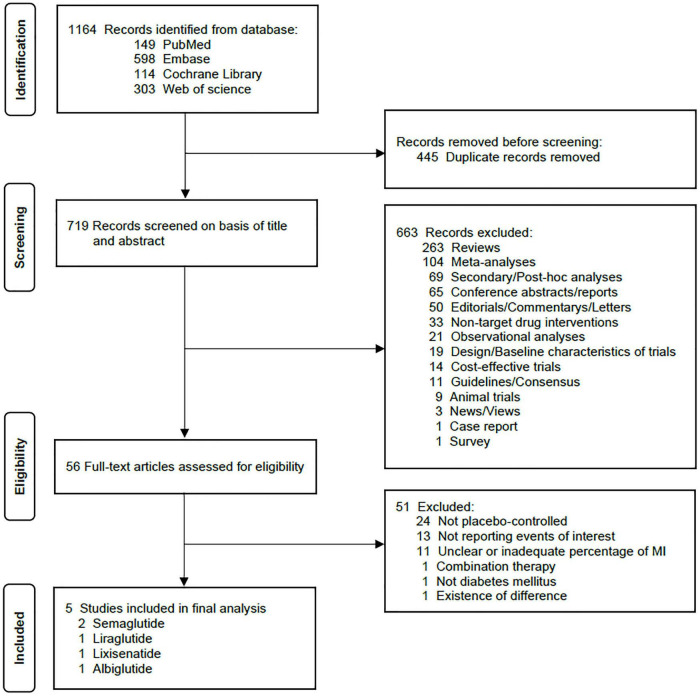
PRISMA flow diagram of study selection. MI, myocardial infarction.

A total of 1,164 citations were retrieved according to the search strategy. After removing duplicates and screening the titles and abstracts, 56 sources were assessed by full-text examination for their potential eligibility. Six trials met our initial criteria. Excluding a trial conducted by Kumarathurai et al. (19). considering that it differed greatly from the other five studies in terms of the number of participants, duration of follow-up, and gender proportion and that it reported only one incident of AF in the placebo arm, we included five trials ultimately (20–24). The flow diagram of study selection is shown in Figure 1.

The total number of participants included was 31,314 (15,655 on GLP-1RAs and 15,659 on placebo), among whom 66.9% were male. The mean age ranged from 60.3 to 66.0 years, and the median follow-up duration ranged from 1.3 to 3.8 years. The GLP-1RAs used were Lixisenatide (one trial, 6,068 patients), albiglutide (one trial, 9,463 patients), Liraglutide (one trial, 9,340 patients) and Semaglutide (two trials, 6,480 patients). Participants included in all studies were T2DM patients, and the proportion of patients combined with MI was > 30%. Characteristics of the included trials in this analysis are depicted in Table 1. All trials had a low risk of bias, as presented in Supplementary Table 2.

**TABLE 1:** Basic characteristics of the included studies (20–24).

Trial	Drug	Median follow-up duration (years)	Total participants (GLP-1 RAs/Placebo)	Male, n (%)	Median age (years)	Patients with a history of MI, n (%)	NCT number
ELIXA (20)	Lixisenatide	2.1	6,068 (3,034/3,034)	4,207 (69.3%)	60.3	5,014 (82.6%)	NCT01147250
HARMOzNY OUTCOMES (21)	Albiglutide	1.6	9,463 (4,731/4,732)	6,569 (69.4%)	64.1	4,459 (47.1%)	NCT02465515
PIONEER 6 (22)	Semaglutide	1.3	3,183 (1,591/1,592)	2,176 (68.4%)	66.0	1,150 (36.1%)	NCT02692716
SUSTAIN 6 (23)	Semaglutide	2.1	3,297 (1,648/1,649)	2,002 (60.7%)	64.6	1,072 (32.5%)	NCT01720446
LEADER (24)	Liraglutide	3.8	9,340 (4,668/4,672)	6,003 (64.3)	64.3	2,864 (30.7%)	NCT01179048

### Atrial arrhythmias events

**FIGURE 2:**
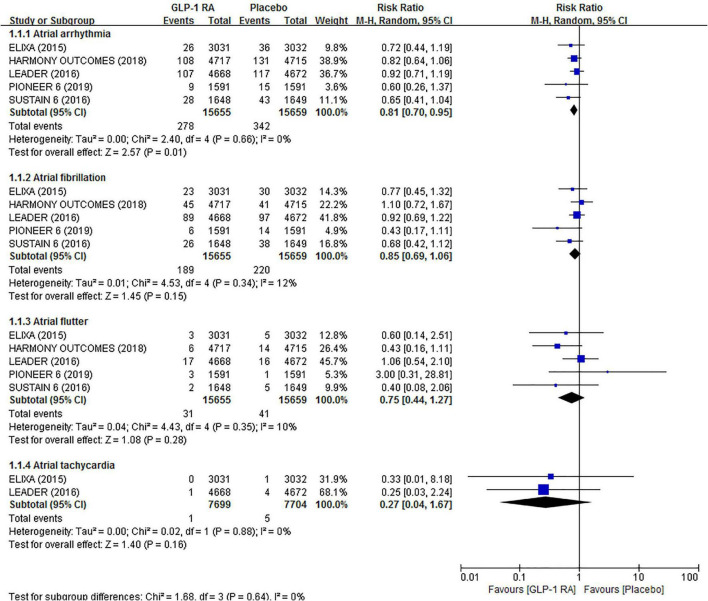
Forest plot of the association between GLP-1RAs and the risk of atrial arrhythmias compared with plabeco.

In this study, atrial arrhythmias were defined as AF, AFL, AF/AFL, and AT. Five trials totally reported 620 events of atrial arrhythmias. The results showed that GLP-1RAs were associated with a 19% risk reduction in atrial arrhythmias (RR 0.81, 95% CI 0.70–0.95) compared to placebo. However, when atrial arrhythmias were subdivided into AF (RR 0.85, 95% CI 0.69–1.06), AFL (RR 0.75, 95% CI 0.44–1.27), and AT (RR 0.27, 95% CI 0.04–1.67) to analyze separately, we found no statistically significant difference between GLP-1RAs and placebo. In all of the above analyses, there was low statistical heterogeneity across trials (*I*^2^ = 0%, *I*^2^ = 12%, *I*^2^ = 10%, and *I*^2^ = 0%, respectively). The related forest plot is shown in Figure 2.

### Ventricular arrhythmias events

**FIGURE 3:**
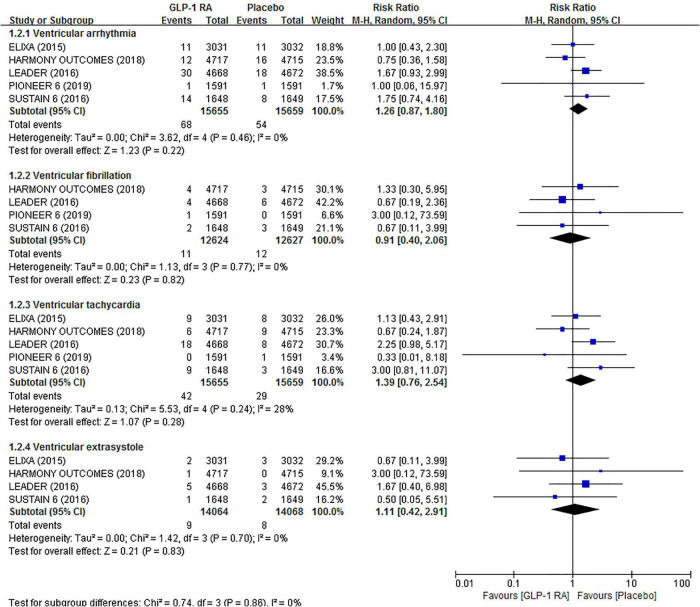
Forest plot of the association between GLP-1RAs and the risk of ventricular arrhythmias compared with plabeco.

In total, 92 events of ventricular arrhythmias, including VF, VT, VE, etc., were reported in all included trials. Pooled analysis showed no significant effect of GLP-1RAs on the risk of ventricular arrhythmias (RR 1.26, 95% CI 0.87–1.80). Likewise, GLP-1RAs did not significantly influence the risk of VF (RR 0.91, 95% CI 0.40–2.06), VT (RR 1.39, 95% CI 0.76–2.54), and VE (RR 1.11, 95% CI 0.42–2.91) compared to placebo in separate analyze. In all of the above analyses, there was low statistical heterogeneity across trials (*I*^2^ = 0%, *I*^2^ = 0%, *I*^2^ = 28%, and *I*^2^ = 0%, respectively). The related forest plot is shown in Figure 3.

### Other arrhythmias

**FIGURE 4:**
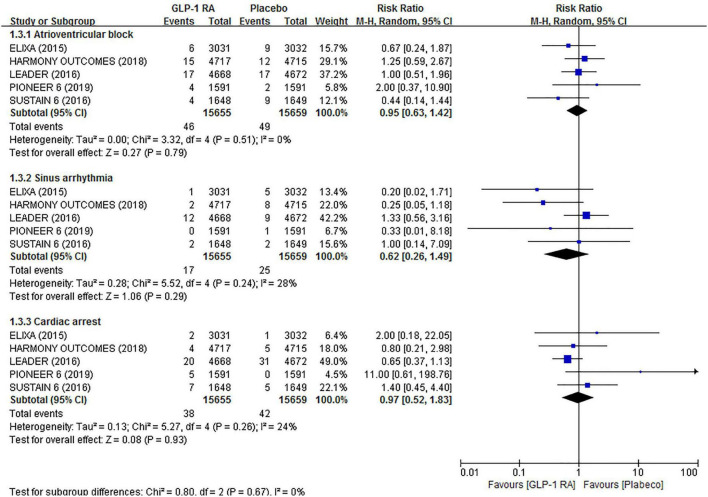
Forest plot of the association between GLP-1RAs and the risk of other arrhythmias compared with plabeco.

For AVB (RR 0.95, 95% CI 0.63–1.42), sinus arrhythmia (RR 0.62, 95% CI 0.26–1.49), and cardiac arrest (RR 0.97, 95% CI 0.52–1.83), no significant associations were observed in the GLP-1RAs arm compared to the placebo arm. There was also low statistical heterogeneity across trials (*I*^2^ = 0%, *I*^2^ = 0%, *I*^2^ = 28%, and *I*^2^ = 0%, respectively) in the above analyses. The related forest plot is shown in Figure 4.

### Subgroups analysis

The results of subgroup analysis based on the drug classes of GLP-1RAs demonstrated that the risk of atrial arrhythmias (RR 0.64, 95% CI 0.42–0.96) and AF (RR 0.62, 95% CI 0.40–0.96) were reduced by 36 and 38%, respectively by Semaglutide, whereas others were not (Table 2). In addition, Albiglutide, Lixisenatide, and Liraglutide did not influence the risk of various arrhythmias (Table 2).

**TABLE 2:** Result of subgroup analysis.

Type of outcome	GLP-1RA agent	Trial	Event/Total of GLP-1RA	Event/Total of Placebo	RR (95% CI)	*P*-value	*I*^2^ of subgroup
Atrial arrhythmia	Lixisenatide	ELIXA	26/3,031	36/3,032	0.72 (0.44, 1.19)	0.20	0%
	Albiglutide	HARMONY OUTCOMES	108/4,717	131/4,715	0.82 (0.64, 1.06)	0.13	
	Liraglutide	LEADER	107/4,668	117/4,672	0.92 (0.71, 1.19)	0.50	
	Semaglutide	PIONEER 6, SUSTAIN 6	37/3,239	58/3,240	0.64 (0.42, 0.96)	0.03	
AF	Lixisenatide	ELIXA	23/3,031	30/3,032	0.77 (0.45, 1.32)	0.34	20.90%
	Albiglutide	HARMONY OUTCOMES	45/4,717	41/4,715	1.10 (0.72, 1.67)	0.67	
	Liraglutide	LEADER	89/4,668	97/4,672	0.92 (0.69, 1.22)	0.56	
	Semaglutide	PIONEER 6, SUSTAIN 6	32/3,239	52/3,240	0.62 (0.40, 0.96)	0.03	
AFL	Lixisenatide	ELIXA	3/3,031	5/3,032	0.60 (0.14, 2.51)	0.48	0%
	Albiglutide	HARMONY OUTCOMES	6/4,717	14/4,715	0.43 (0.16, 1.11)	0.08	
	Liraglutide	LEADER	17/4,668	16/4,672	1.06 (0.54, 2.10)	0.86	
	Semaglutide	PIONEER 6, SUSTAIN 6	5/3,239	6/3,240	0.94 (0.13, 6.59)	0.95	
AT	Lixisenatide	ELIXA	0/3,031	1/3,032	0.33 (0.01, 8.18)	0.50	0%
	Liraglutide	LEADER	1/4,668	4/4,672	0.25 (0.03, 2.24)	0.22	
Ventricular arrhythmia	Lixisenatide	ELIXA	11/3,031	11/3,032	1.00 (0.43, 2.30)	1.00	13.70%
	Albiglutide	HARMONY OUTCOMES	12/4,717	16/4,715	0.75 (0.36, 1.58)	0.45	
	Liraglutide	LEADER	30/4,668	18/4,672	1.67 (0.93, 2.99)	0.09	
	Semaglutide	PIONEER 6, SUSTAIN 6	15/3,239	9/3,240	1.67 (0.73, 3.81)	0.23	
VF	Albiglutide	HARMONY OUTCOMES	4/4,717	3/4,715	1.33 (0.30, 5.95)	0.71	0%
	Liraglutide	LEADER	4/4,668	6/4,672	0.67 (0.19, 2.36)	0.53	
	Semaglutide	PIONEER 6, SUSTAIN 6	3/3,239	3/3,240	0.95 (0.20, 4.54)	0.95	
VT	Lixisenatide	ELIXA	9/3,031	8/3,032	1.13 (0.43, 2.91)	0.81	12.50%
	Albiglutide	HARMONY OUTCOMES	6/4,717	9/4,715	0.67 (0.24, 1.87)	0.44	
	Liraglutide	LEADER	18/4,668	8/4,672	2.25 (0.98, 5.17)	0.06	
	Semaglutide	PIONEER 6, SUSTAIN 6	9/3,239	4/3,240	1.66 (0.24, 11.25)	0.61	
VE	Lixisenatide	ELIXA	2/3,031	3/3,032	0.67 (0.11, 3.99)	0.66	0%
	Albiglutide	HARMONY OUTCOMES	1/4,717	0/4,715	3.00 (0.12, 73.59)	0.50	
	Liraglutide	LEADER	5/4,668	3/4,672	1.67 (0.40, 6.98)	0.48	
	Semaglutide	SUSTAIN 6	1/1,648	2/1,649	0.50 (0.05, 5.51)	0.57	
Atrioventricular block	Lixisenatide	ELIXA	6/3,031	9/3,032	0.67 (0.24, 1.87)	0.44	0%
	Albiglutide	HARMONY OUTCOMES	15/4,717	12/4,715	1.25 (0.59, 2.67)	0.56	
	Liraglutide	LEADER	17/4,668	17/4,672	1.00 (0.51, 1.96)	1.00	
	Semaglutide	PIONEER 6, SUSTAIN 6	8/3,239	11/3,240	0.83 (0.19, 3.54)	0.80	
Sinus arrhythmia	Lixisenatide	ELIXA	1/3,031	5/3,032	0.20 (0.02, 1.71)	0.14	41.20%
	Albiglutide	HARMONY OUTCOMES	2/4,717	8/4,715	0.25 (0.05, 1.18)	0.08	
	Liraglutide	LEADER	12/4,668	9/4,672	1.33 (0.56, 3.16)	0.51	
	Semaglutide	PIONEER 6, SUSTAIN 6	2/3,239	3/3,240	0.74 (0.14, 3.94)	0.73	
Cardiac arrest	Lixisenatide	ELIXA	2/3,031	1/3,032	2.00 (0.18, 22.05)	0.57	0%
	Albiglutide	HARMONY OUTCOMES	4/4,717	5/4,715	0.80 (0.21, 2.98)	0.74	
	Liraglutide	LEADER	20/4,668	31/4,672	0.65 (0.37, 1.13)	0.13	
	Semaglutide	PIONEER 6, SUSTAIN 6	12/3,239	5/3,240	2.60 (0.38, 17.90)	0.33	

RR, risk ratio; 95% CI, 95% confidence interval; AF, atrial fibrillation; AFL, atrial flutter; AT, atrial tachycardia; VF, ventricular fibrillation; VT, ventricular tachycardia; VE, ventricular extrasystoles.

### Sensitivity analysis

In the sensitivity analyses using the fixed-effects models or OR, the results of the association between GLP-1RAs and arrhythmias changed slightly. Still, they did not affect the original conclusions (Supplementary Table 3).

## Discussion

Our systematic review and meta-analysis of 5 trials with 31,314 patients found that treatment with GLP-1RAs might be associated with a lower risk of atrial arrhythmias. In subgroup analysis, we observed that Semaglutide reduced the risk of atrial arrhythmias and AF, while no anti-arrhythmic effect was revealed for the other GLP-1RAs.

A previous meta-analysis assessing the association between GLP-1RAs and arrhythmias in patients with T2DM did not find that the use of GLP-1RAs was associated with a reduced risk of atrial arrhythmias (OR 0.96, 95% CI 0.869–1.066; *P* = 0.4) (11). Nevertheless, our study identified this association through the meta-analysis of included studies that met the critical criterion of a proportion of patients with T2DM and MI > 30%.

Our results suggest that the anti-atrial arrhythmia effect of GLP-1RAs seems to be more pronounced for patients with T2DM combined with MI. The mechanisms may be as follows. Elevated left ventricular end-diastolic pressure (LVEDP) after MI leads to increased left atrial (LA) pressure and LA volume, which stretches the LA wall. As a result, the surface area and oxidative stress of LA increased. The increased surface area may alter the conduction of LA electrical activity and thus contribute to the initiation and maintenance of arrhythmias (25). Wohlfart et al. randomly divided rats into Lixisenatide or placebo groups over ten weeks one day after ligating the left coronary artery of rats reversibly for 30 min. They found that Lixisenatide significantly reduced cardiac LVEDP in rats compared to placebo (26). In addition, reversing LA stretch by mechanical left ventricle (LV) unloading reduces phosphorylation of ryanodine receptor (RyR) at PKA dependent and CAMK-II dependent sites, thereby reducing oxidative stress and exerting an anti-atrial arrhythmias effect (25). Research has shown that Exendin-4 suppressed CaMK-II activity and reduced cardiac RyR phosphorylation in a rat model of MI (27). The clinical study also indicated Liraglutide reduced LV diastolic filling and LV filling pressure and unloaded the LV (28). The other mechanisms may involve inhibition of the PI3K/AKT signaling pathway and attenuation of atrial fibrosis yet (14).

Although we could not confirm that GLP-1RAs reduced the risk of AF to the conventional level of statistical significance in a separate analysis, the result might detect a favorable signal toward AF reduction in the GLP-1RAs group (*P* = 0.15). Some previous studies have suggested that Albiglutide may be associated with an increased risk of AF (8, 29). Therefore, we tried to exclude the Harmony outcomes trial of Albiglutide and then analyzed it again. Interestingly enough, the difference between relevant groups was significantly greater than before (RR 0.81, 95% CI 0.65–1.01; *p* = 0.06), which did not meet the statistical significance by only a tiny margin (Supplementary Figure 1A). The analysis’s result further reached the critical values when we changed the analysis to the fixed-effects models (RR 0.81, 95% CI 0.65–1.00; *p* = 0.05), as shown in Supplementary Figure 1B. Significantly, our subgroup analysis indicated that Semaglutide was associated with a reduced risk of AF. The above may illustrate differences existing in different drug classes of GLP-1RAs concerning their anti-arrhythmic effects.

The results of separate analyses failed to detect a significant association between GLP-1RAs and lower risk of AFL and AT as well. However, relative to AF, the number of AFL or AT events in the included trials of this study were relatively few leading to a wide confidence interval. In other words, our results might have been influenced by low power so that they were not statistically significant. Larger RCTs are needed to evaluate the association between GLP-1RAs and these atrial arrhythmias before reaching definitive conclusions.

Data from animal studies have suggested that GLP-1RAs might exert beneficial effects on ventricular arrhythmias after MI. It is well known that sympathetic nervous system (SNS) overactivity is a common condition after MI, producing certain cardiac toxicity while playing a partial compensatory role. This leads to complications such as ventricular arrhythmias (30). The reducing effect of GLP-1RAs on ventricular arrhythmias under the condition of post-MI SNS activation was confirmed in a rat study, which might be mediated indirectly by acetylcholine and nitric oxide (NO) (31). Another animal study showed that Exendin-4 could attenuate ventricular arrhythmias caused by ischemia in rats via mitochondrial K_*ATP*_ channels of which the activation are able to reduce mitochondrial calcium overload and maintain mitochondrial calcium homeostasis during MI (15).

However, no protective effect of GLP-1RAs against ventricular arrhythmias was observed in our meta-analysis. This indicates that the above favorable mechanisms of GLP-1RAs on ventricular arrhythmias might be negligible or slight overall for humans, or might be diminished by other reasons. Thus, these mechanisms may be ineffective clinically. The research of Zhong et al. may confirm our conjecture (32). Post-MI LV remodeling, a pathological change including cardiac structure and function, is closely associated with developing ventricular arrhythmias after MI (33, 34). It’s been proven that Liraglutide improved cardiac structure parameters such as left ventricular end-diastolic diameter (LVEDD) and left ventricular posterior wall thickness (LVPWT) (35). In the study of Zhong et al., though, Liraglutide did not improve the left ventricular ejection fraction (LVEF), the early filling velocity on transmittal Doppler/early relaxation velocity on tissue Doppler (E/e’) and other parameters of left ventricular systolic or diastolic function (32). Consequently, the effects of GLP-1RAs on LV remodeling might be moderate overall, and therefore its anti-ventricular arrhythmias potential is negligible. In addition, GLP-1RAs have been shown to cause an increase in heart rate by stimulating SNS, but the theoretical higher cardiovascular risk was not found in multiple studies related to GLP-1RAs (36). Another potential explanation for the inconsistent conclusions between the animal studies and this meta-analysis on ventricular arrhythmias may be that the trials included in our study only had a high proportion of T2DM patients combined with MI. That is to say, MI is not present in all T2DM patients included in this meta-analysis, meaning the indirect nature of our study.

Currently, studies on the association among GLP-1RAs and AVB, sinus arrhythmias, and cardiac arrest are rare relatively. In our research, we found no correlation. However, this conclusion was limited by the small number of events, and future larger prospective studies are needed to confirm their specific association.

This meta-analysis has several limitations. First, as there were few RCTs to directly study the efficacy of GLP-1RAs compared to placebo in patients with T2DM combined with MI, we tried to verify our purpose by indirectly analyzing RCTs with a high proportion of target patients. Second, part of included patients also combined with heart failure, kidney disease, etc., and thus the heterogeneity of such population may have influenced the final results. Third, all included studies reported arrhythmia events as SAEs, rather than the pre-specified outcomes. Therefore, no systematic measure to evaluate for arrhythmias was described. This may lead to some measurement bias. Fourth, the number of some arrhythmia events in this study was relatively few leading to wide confidence intervals, which might influence our results.

## Conclusion

To the authors’ knowledge, this is the first systematic review and meta-analysis to assess the relationship between GLP-1RAs and arrhythmias in patients with T2DM and MI. In summary, the results suggest that GLP-1RAs may be associated with reduced risk for atrial arrhythmias. Furthermore, the anti-atrial arrhythmia effect of GLP-1RAs seems more significant for patients with T2DM combined with MI. In addition, the difference appears to exist in different drug classes of GLP-1RAs concerning their anti-arrhythmic effects. More prospective studies are needed to clarify the definitive anti-arrhythmic role of this drug and its heterogeneous effect in different drug classes.

## Data availability statement

The original contributions presented in this study are included in the article/Supplementary material, further inquiries can be directed to the corresponding author/s.

## Author contributions

ZL, NB, and SW: literature database search and writing of the manuscript. ZL and YF: study selection, data extraction, and quality assessment. ZL, NB, SW, HL, and JY: data analysis of the results. DC and JG: conception, design, and revision of the manuscript. All authors contributed to the article and approved the submitted version.
