# Supplementary material for: A meta-analysis evaluating indirectly GLP-1 receptor agonists and arrhythmias in patients with type 2 diabetes and myocardial infarction
Source: Front Cardiovasc Med. 2022 Oct 5;9:1019120. doi: 10.3389/fcvm.2022.1019120 (PMC9581215; doi:10.3389/fcvm.2022.1019120)
Supplement: Supplementary file 1 [file Data_Sheet_1.PDF]

Supplementary Material Table S1 Search strategy.

Patient	#1	"diabetes mellitus"[Mesh] OR "diabetes mellitus, Type 2"[Mesh]
	#2	"diabetes mellitus"
	#3	"myocardial infarction"[Mesh]
	#4	"myocardial infarction" OR "myocardial infarctions" OR "cardiovascular stroke" OR "cardiovascular strokes" OR "myocardial infarct" OR "myocardial infarcts" OR "heart attack" OR "heart attacks"
	#5	"ST elevation myocardial infarction"[Mesh] OR "Non-ST elevated myocardial infarction"[Mesh]
	#6	"STEMI" OR "ST elevation myocardial infarction" OR "ST segment elevation myocardial infarction" OR "ST elevated myocardial infarction"
	#7	"NSTEMI" OR "Non-ST elevated myocardial infarction" OR "Non ST elevated myocardial infarction" OR "Non-ST-Elevation myocardial infarction" OR "Non ST elevation myocardial infarction" OR "Non-ST-Elevation myocardial infarctions"
	#8	(#1 OR #2) AND (#3 OR #4 OR #5 OR #6 OR #7)
Intervention	#9	"Glucagon-Like Peptide 1"[Mesh] OR "Glucagon-Like Peptide-1 Receptor"[Mesh]
	#10	"Glucagon-Like Peptide 1" OR "Glucagon Like Peptide 1" OR "GLP-1" OR "GLP 1" OR "Glucagon-Like Peptide-1"
	#11	"Glucagon-Like Peptide-1 Receptor" OR "Glucagon Like Peptide 1 Receptor" OR "Peptide-1 Receptor, Glucagon-Like" OR "Receptor, Glucagon-Like Peptide-1" OR "GLP-1R Receptor" OR "GLP 1R Receptor" OR "Receptor, GLP-1R" OR "GLP1R Protein" OR "Protein, GLP1R" OR "GLP-1 Receptor" OR "GLP 1 Receptor" OR "Receptor, GLP-1" OR "GLP1R Receptor"
	#12	"*glutide" OR "*natide"
	#13	"Exenatide" [Mesh] OR "Liraglutide" [Mesh]
	#14	"Dulaglutide"[Supplementary Concept] OR "Lixisenatide"[Supplementary Concept] OR "Semaglutide"[Supplementary Concept]
	#15	"rGLP-1 protein"[Supplementary Concept]
	#16	"Albiglutide" OR "Exenatide" OR "Exendin 4" OR "Exendin-4" OR "Liraglutide" OR "Dulaglutide" OR "Lixisenatide" OR "Semaglutide"
	#17	#9 OR #10 OR #11 OR #12 OR #13 OR #14 OR #15 OR #16
Type of study	#18	"randomized controlled trial"[Publication Type]
	#19	"randomized controlled trial" OR "randomi?ed" OR "random*"
	#20	#18 OR #19
Combined	#21	#8 AND #17 AND #20

Supplementary Material Table S2 Risk of bias assessment.

Trial name	Random sequence generation	Allocation concealment	Blinding of participant and personnel	Blinding of outcome assessment	Incomplete outcome data	Selective reporting	Other biases	Overall risk of bias
ELIXA (2015)	Low	Low	Low	Low	Low	Low	Low	Low
HARMONY OUTCOMES (2018)	Low	Low	Low	Low	Low	Low	Low	Low
PIONEER 6 (2019)	Low	Low	Low	Low	Low	Low	Low	Low
SUSTAIN 6 (2016)	Low	Low	Low	Low	Low	Low	Low	Low
LEADER (2016)	Low	Low	Low	Low	Low	Low	Low	Low

Supplementary Material Table S3 Result of sensitivity analysis.

Type of outcome	Sensitivity analysis	RR (95% CI)	P value
Atrial arrhythmia	Primary analysis	0.81 (0.70, 0.95)	0.01
	Transformed effect measure to OR	0.81 (0.69, 0.95)	0.01
	Transformed the analsis to fixed-effects model	0.81 (0.70, 0.95)	0.01
AF	Primary analysis	0.85 (0.69, 1.06)	0.15
	Transformed effect measure to OR	0.85 (0.69, 1.06)	0.15
	Transformed the analsis to fixed-effects model	0.86 (0.71, 1.04)	0.12
AFL	Primary analysis	0.75 (0.44, 1.27)	0.28
	Transformed effect measure to OR	0.75 (0.44, 1.27)	0.28
	Transformed the analsis to fixed-effects model	0.76 (0.47, 1.21)	0.24
AT	Primary analysis	0.27 (0.04, 1.67)	0.16
	Transformed effect measure to OR	0.27 (0.04, 1.67)	0.16
	Transformed the analsis to fixed-effects model	0.27 (0.04, 1.66)	0.16
Ventricular arrhythmia	Primary analysis	1.26 (0.87, 1.80)	0.22
	Transformed effect measure to OR	1.26 (0.87, 1.81)	0.22
	Transformed the analsis to fixed-effects model	1.26 (0.88, 1.80)	0.20
VF	Primary analysis	0.91 (0.40, 2.06)	0.82
	Transformed effect measure to OR	0.91 (0.40, 2.06)	0.82
	Transformed the analsis to fixed-effects model	0.92 (0.41, 2.05)	0.84
VT	Primary analysis	1.39 (0.76, 2.54)	0.28
	Transformed effect measure to OR	1.39 (0.76, 2.55)	0.28
	Transformed the analsis to fixed-effects model	1.44 (0.90, 2.30)	0.13
VE	Primary analysis	1.11 (0.42, 2.91)	0.83
	Transformed effect measure to OR	1.11 (0.42, 2.91)	0.83
	Transformed the analsis to fixed-effects model	1.12 (0.44, 2.82)	0.81
Atrioventricular block	Primary analysis	0.95 (0.63, 1.42)	0.79
	Transformed effect measure to OR	0.95 (0.63, 1.42)	0.79
	Transformed the analsis to fixed-effects model	0.94 (0.63, 1.40)	0.76
Sinus arrhythmia	Primary analysis	0.62 (0.26, 1.49)	0.29
	Transformed effect measure to OR	0.62 (0.26, 1.49)	0.29
	Transformed the analsis to fixed-effects model	0.69 (0.37, 1.26)	0.23
Cardiac arrest	Primary analysis	0.97 (0.52, 1.83)	0.93
	Transformed effect measure to OR	0.97 (0.52, 1.84)	0.93
	Transformed the analsis to fixed-effects model	0.91 (0.59, 1.40)	0.66

Abbreviations: RR, risk ratio; OR, odds ratio; 95% CI, 95% confidence interval; AF, atrial fibrillation; AFL, atrial flutter; AT, atrial tachycardia; VF, ventricular fibrillation; VT, ventricular tachycardia; VE, ventricular extrasystoles.

a

Study or Subgroup	GLP-1 RA Events	GLP-1 RA Total	Placebo Events	Placebo Total	Weight	Risk Ratio M-H, Random, 95% CI
ELIXA (2015)	23	3031	30	3032	16.3%	0.77 [0.45, 1.32]
HARMONY OUTCOMES (2018)	45	4717	41	4715	0.0%	1.10 [0.72, 1.67]
LEADER (2016)	89	4668	97	4672	58.9%	0.92 [0.69, 1.22]
PIONEER 6 (2019)	6	1591	14	1591	5.2%	0.43 [0.17, 1.11]
SUSTAIN 6 (2016)	26	1648	38	1649	19.6%	0.68 [0.42, 1.12]
**Total (95% CI)**		**10938**		**10944**	**100.0%**	**0.81 [0.65, 1.01]**
Total events	144		179			

Heterogeneity: Tau² = 0.00; Chi² = 2.94, df = 3 (P = 0.40); I² = 0%
Test for overall effect: Z = 1.90 (P = 0.06)

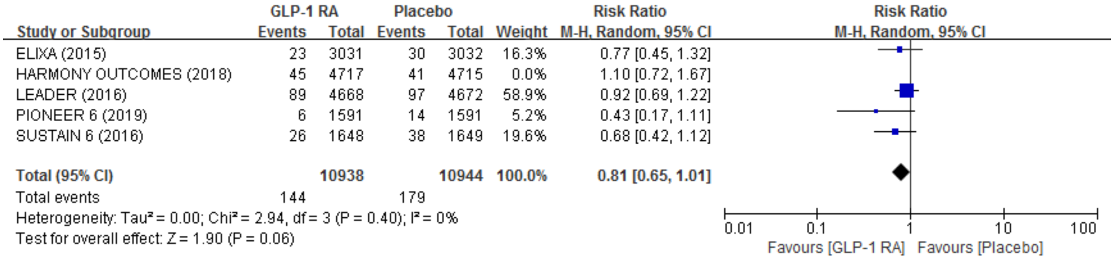

b

Study or Subgroup	GLP-1 RA Events	GLP-1 RA Total	Placebo Events	Placebo Total	Weight	Risk Ratio M-H, Fixed, 95% CI
ELIXA (2015)	23	3031	30	3032	16.8%	0.77 [0.45, 1.32]
HARMONY OUTCOMES (2018)	45	4717	41	4715	0.0%	1.10 [0.72, 1.67]
LEADER (2016)	89	4668	97	4672	54.2%	0.92 [0.69, 1.22]
PIONEER 6 (2019)	6	1591	14	1591	7.8%	0.43 [0.17, 1.11]
SUSTAIN 6 (2016)	26	1648	38	1649	21.2%	0.68 [0.42, 1.12]
**Total (95% CI)**		**10938**		**10944**	**100.0%**	**0.81 [0.65, 1.00]**
Total events	144		179			

Heterogeneity: Chi² = 2.94, df = 3 (P = 0.40); I² = 0%
Test for overall effect: Z = 1.95 (P = 0.05)

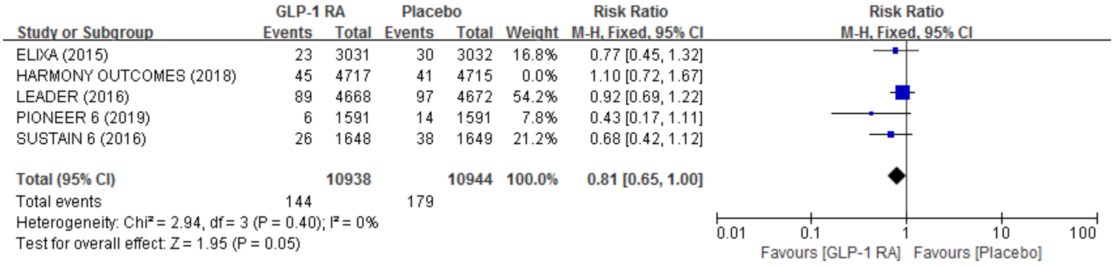

Supplementary Material Figure S1 Forest plot of the association between GLP-1RAs and the risk of atrial fibrillation compared with plabeco after removing Harmony outcomes trail.
